# Which is better for gastric cancer patients, perioperative or adjuvant chemotherapy: a meta-analysis

**DOI:** 10.1186/s12885-016-2667-5

**Published:** 2016-08-12

**Authors:** Jun-hua Zhao, Peng Gao, Yong-xi Song, Jing-xu Sun, Xiao-wan Chen, Bin Ma, Yu-chong Yang, Zhen-ning Wang

**Affiliations:** Department of Surgical Oncology and General Surgery, the First Hospital of China Medical University, Shenyang, 110001 People’s Republic of China

**Keywords:** Gastric cancer, Perioperative chemotherapy, Adjuvant chemotherapy, Overall survival, Combination chemotherapy

## Abstract

**Background:**

The preferred chemotherapy method for gastric cancer continues to be matter of debate. We performed a meta-analysis to comparing prognosis and safety between perioperative chemotherapy and adjuvant chemotherapy to identify the better chemotherapy option for gastric cancer.

**Methods:**

We searched the PubMed, EMBASE, Cochrane Library, and Ovid databases for eligible studies until February 2016. The main endpoints were prognostic value (hazard ratio [HR] for overall survival [OS] and 1-, 2-, 3-, and 5-year survival rate), response rate of chemotherapy, radical resection rate, post-operative complication rate, and adverse effects of chemotherapy.

**Results:**

Five randomized controlled trials and six clinical controlled trials involving 1,240 patients were eligible for analysis. Compared with the adjuvant chemotherapy group, the perioperative chemotherapy group had significantly better prognosis (HR, 0.74; 95 % CI, 0.61 to 0.89; *P* < 0.01). The difference between the two groups remained significant in the studies that used combination chemotherapy as the neoadjuvant chemotherapy regimen (HR, 0.59; 95 % CI, 0.46 to 0.76; *P* < 0.01) but were not significant in the studies that used fluoropyrimidine monotherapy (HR, 0.93; 95 % CI, 0.56 to 1.55; *P* = 0.84). Furthermore, the two groups showed no significant differences in the post-operative complication rates (relative risk, 0.98; 95 % CI, 0.63 to 1.51; *P* = 0.91) or adverse effects of chemotherapy (*P* > 0.05 for all adverse effects).

**Conclusion:**

Perioperative chemotherapy showed improved survival compared to adjuvant chemotherapy for gastric cancer. In addition, combination chemotherapy resulted in better survival compared to monotherapy in the neoadjuvant chemotherapy regimens.

**Electronic supplementary material:**

The online version of this article (doi:10.1186/s12885-016-2667-5) contains supplementary material, which is available to authorized users.

## Background

Gastric cancer (GC) is the fourth most common cancer and the second leading cause of cancer-related deaths worldwide [1, 2]. To date, surgery is the only curative treatment for GC. However, the results are still unsatisfactory, owing to the high rate of metastasis and relapse [1, 3].

Chemotherapy together with surgery has shown promising results. For instance, a randomized controlled trial conducted by Cunningham et al. [4] showed that perioperative chemotherapy (PC) could result in better survival than surgery alone. Similarly, Bang et al. [5] showed that adjuvant chemotherapy (AC) could improve survival over surgery alone. However, the method of delivery of chemotherapy for GC is still a matter of debate. PC consists of preoperative (neoadjuvant) chemotherapy and postoperative chemotherapy, and is provided as standard of care in NCCN guideline for GC. At the same time, the application of AC is limited to situations where neoadjuvant therapy had not been given prior to surgery [6]. However chemotherapy given prior to surgery may reduce tumor burden and eradicate micrometastatic foci outside the surgical field [7, 8]. Several studies have emphasized the survival benefits of PC to the patients [9, 10]. However, chemortherapy given prior to surgery can cause fibrosis and tissue edema, which may cause difficulties during surgery [11], causing adverse effects to the patients [12, 13]. Therefore, we performed a meta-analysis to compare the prognostic value, side effects, and post-operative complications of PC and AC in patients with GC.

## Methods

### Search strategy

Studies were selected by searching major medical databases (PubMed, EMBASE, Cochrane Library, and Ovid) for all articles published until February 1, 2016. We used the following keywords: “neoadjuvant”, “preoperative”, “perioperative”, “chemotherapy”, “stomach neoplasm”, “gastric cancer”, and “gastrectomy” Then, we narrowed the search by browsing the abstracts, methods, and references of the articles retrieved.

### Inclusion and exclusion criteria

The studies that met the following criteria were included: (i) publications that compared PC with AC in patients with GC undergoing surgery; (ii) the full text of the articles was available, with a clear description of the chemotherapy regimens used in the study; (iii) at least one of the outcome measures mentioned below was reported or could be calculated from the data provided. In cases of overlap between authors or institutions, only the higher-quality or more recent study was selected. Studies were excluded for the following reasons: (i) PC and AC were not compared in the patients with GC; (ii) post-operative chemotherapy was not applied in either the PC or AC groups; (iii) radiotherapy was part of treatment.

### Outcome measures, data extraction, and assessment of the risk of bias

The primary outcomes were prognostic value (hazard ratio [HR] for overall survival [OS] and 1-, 2-, 3-, and 5-year survival rate), response rate of chemotherapy (response rate: complete response [CR] or partial response [PR] after chemotherapy), radical resection rate; total post-operative complication rate (defined on the basis of the system for reporting complications established by the Memorial Sloan-Kettering Cancer Center [14]), and the adverse effects of chemotherapy. Two authors independently extracted data from full-text articles using a unified datasheet. Randomized controlled trials (RCTs) were evaluated using the Jadad Composite Scale (JCS), wherein high-quality trials should score ≥ 3 of a maximum possible score of 5. Controlled clinical trials (CCTs) were evaluated using the Newcastle–Ottawa Scale [15], wherein high-quality trials should score ≥ 7 of a maximum possible score of 9, and moderate-quality trials should score ≥ 5. Disagreements were presented to a third author and resolved by discussions among the investigators.

### Statistical analysis

This meta-analysis was conducted using RevMan software version 5.2 (Cochrane Collaboration). The risk ratio (RR) and HR were used to evaluate the prognostic effect. If the HR and its variance were not reported directly in the original study, these values were calculated using a software designed by Tierney et al. [16]. For HR, we performed subgroup analysis based on available method, such as study design, NAC regimen, et al. In addition, the RR was used to analyze other discontinuous variables. Both ratios were reported with 95 % confidence intervals (CIs). Heterogeneity was determined using the *χ*^2^ test or Cochran Q test. I^2^ was used to quantify heterogeneity. *P* < 0.10 and I^2^ > 50 % indicated significant heterogeneity. The inverse variance method with a fixed-effects model was applied when heterogeneity was not found, whereas the random-effects model was used when heterogeneity was found. Publication bias was tested using funnel plots. *P* < 0.05 was considered significant when measuring the effect sizes. This manuscript reporting adheres to PRISMA guidelines for reporting systematic reviews and meta-analyses.

## Results

### Eligible studies

**Fig. 1:**
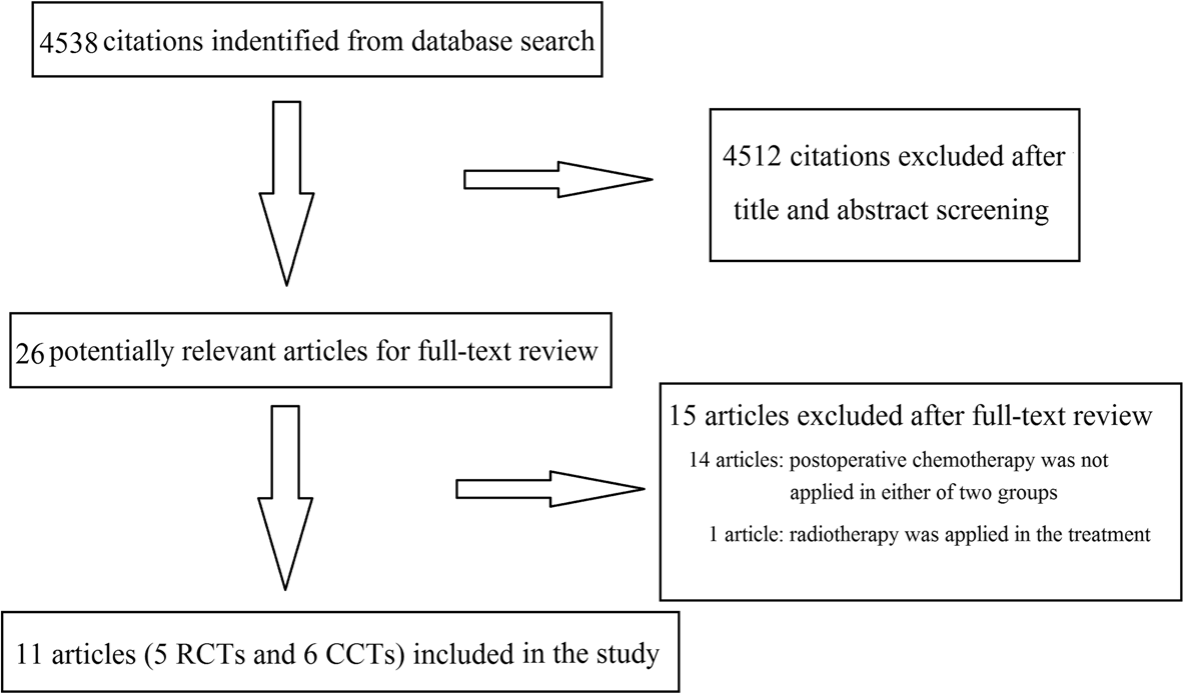
Flow chart of articles selection

The search for the aforementioned keywords allowed the identification of 4538 articles. Five RCTs [17–21] and six CCTs [9, 11, 22–25] were considered eligible for this meta-analysis (Fig. 1). The analyses included 1240 patients who were in the PC group (*n* = 557) or in the AC group (*n* = 683). The detailed characteristics of the patients are listed in Table 1 and Additional file 1. Four RCTs scored 3 in the JCS, indicating that they were high-quality studies (Table 2). Three CCTs scored 6 (moderate-quality study) on the Newcastle–Ottawa scale and 3 CCTs scored 7 (high-quality study) (Table 3).

**Table 1:** Main characteristics of including studies

study	year	place	design	Patients number		follow-up	Regimen^a^			Age		Sex (male/female)	
				PC	AC		PC group		AC group	PC	AC	PC	NC
							preoperative	postoperative					
Yonemura	1993	Japan	RCT	29	26	3 years	PMUE	PMUE	PMUE	61.4 ± 8.34	56.4 ± 9.6	21/8	20/6
Kobayashi	2000	Japan	RCT	91	80	5 years	5′-DFUR	5′-DFUR + MMC	5′-DFUR + MMC	57.8	60.2	65/26	55/25
Nio	2004	Japan	RCT	102	193	7 years	UFT	UFT or FPEPIR + UFT	UFT or FPEPIR + UFT	63.5 ± 11.9	65.3 ± 11.5	70/32	141/52
Qu	2010	China	RCT	39	39	≥2 years	PTX + FOLFOX4	PTX + FOLFOX4 or ECF	PTX + FOLFOX4	NA	NA	26/13	22/17
X.Sun	2011	China	RCT	29	26	3 years	DCF	DCF	DCF	52.6 (33–72)		37/18	
Z.Sun	2014	China	CCT	23	35	3 years	FOLFOX4	FOLFOX	FOLFOX	58 (34–79)	57 (31–80)	15/8	22/13
Feng	2015	China	CCT	80	90	Till discharge	SOX	SOX	SOX	61 (21–74)	59 (29–82)	63/17	71/19
Li	2012	China	CCT	33	37	≥ 5 years	FOLFOX	FOLFOX	FOLFOX	65 (41–75)	61 (27–78)	23/10	30/7
J.Zhang	2012	China	CCT	38	42	5 years	mFOLFOX7	mFOLFOX7 or mECF	mFOLFOX7	NA	NA	22/16	26/16
Nishioka	1982	Japan	CCT	64	59	5 years	5-FU	5-FU and MMP	5-FU and MMP	NA	NA	NA	NA
C.Zhang	2004	China	CCT	29	56	5 years	FAP or FMP	FAP or FMP	FAP or FMP	54.9 ± 12.9		69/22	

*PC* perioperative chemotherapy, *AC* adjuvant chemotherapy, *RCT* randomized controlled trails, *CCT* clinical controlled trails

^a^concrete information of regimens is shown in Additional file 1

**Table 2:** The risk of bias of RCTS (Jadad scale)

Reference	Randomization	Blinding	Withdraw and dropout	Jadad’s score	Quality
Yonemura	2	0	0	2	Moderate
Kobayashi	2	0	1	3	High
Nio	2	0	1	3	High
Qu	2	0	1	3	High
X.Sun	2	0	1	3	High

Randomization: randomization was described with appropriate method- 2 score, randomization was described without appropriate method- 1 score, no randomization- 0 score

Blinding: blinding was performed on all doctors and patients- 2 score, blinding was partially performed on doctors and patients- 1 score, no blinding- 0 score

Withdraw and dropout: the reason of withdraw and dropout was described- 1 score, the reason of withdraw and dropout was not described- 0 score

Quality: High-quality trials should score ≥ 3. moderate-quality trials should score ≥ 2

**Table 3:** The risk of bias of CCTS (NOS)

Reference	Selection				Comparability		Outcome			Total	Quality
	REC	SNEC	AE	DO	SC	AF	AO	FU	FUO		
C.Zhang	1	0	1	1	0	0	1	1	1	6	Moderate
Z.Sun	1	1	1	1	0	0	1	1	1	7	High
Feng	1	1	1	1	0	0	1	0	1	6	Moderate
Li	1	1	1	1	0	0	1	0	1	6	Moderate
J.Zhang	1	1	1	1	0	0	1	1	1	7	High
Nishioka	1	1	1	1	0	0	1	1	1	7	High

*REC* representativeness of the exposed cohort, *SNEC* selection of the non-exposed cohort, *AE* ascertainment of exposure, *DO* demonstration that outcome of interest was not present at start of study, *SC* study controls for age, sex, *AF* study controls for any additional factors, *AO* assessment of outcome, *FU* follow-up long enough for outcomes to occur, *FUO* adequacy of follow-up of cohorts

### Hazard ratio for the overall survival

**Fig. 2:**
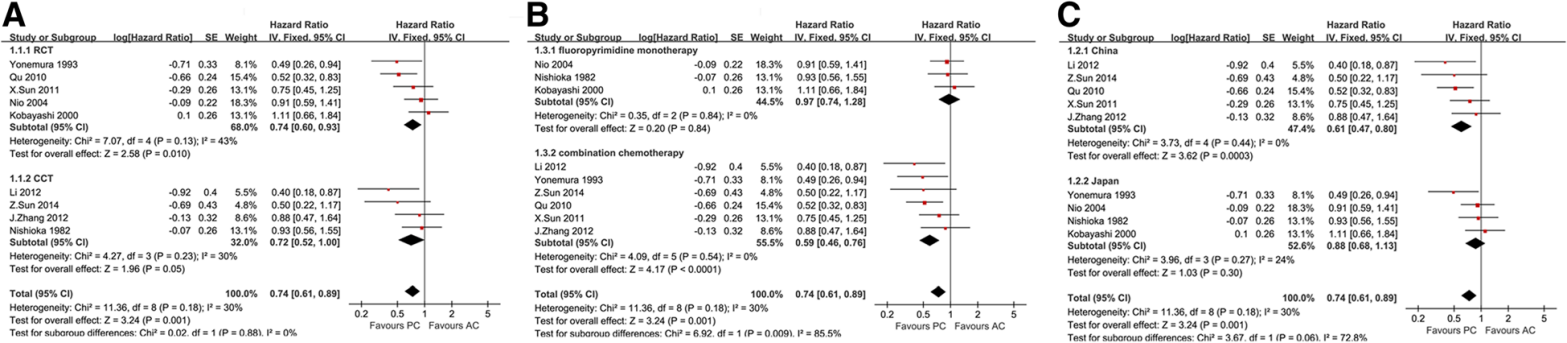
**a**-**c** Meta-analysis of hazard ratio for overall survival subgrouped by (**a**) RCT or CCT, **b** neoadjuvant chemotherapy regimen, (**c**) where the study from

Nine [9, 17–22, 24, 25] out of eleven studies (5 RCTs and 4 CCTs) evaluated provided effective data for the calculation of the HR for OS. Compared with the AC group, the PC group had significantly better prognosis (HR, 0.74; 95 % CI, 0.61 to 0.89; *P* < 0.01, Fig. 2a-c). The difference between the two groups remained significant subgroup analysis that only consisted of RCTs (HR, 0.74; 95 % CI, 0.60 to 0.93; *P* = 0.01) (Fig. 2a).

We also performed a subgroup analysis examining the nine studies divided into two subgroups: the fluoropyrimidine monotherapy subgroup and combination chemotherapy subgroup (Fig. 2b). PC did not demonstrate improved prognosis in the fluoropyrimidine monotherapy subgroup (HR, 0.93; 95 % CI, 0.56 to 1.55; *P* = 0.84) but did show improved prognosis in the combination chemotherapy subgroup (HR, 0.59; 95 % CI, 0.46 to 0.76; *P* < 0.01).

We also performed a subgroup analysis considering the study locations (Fig. 2c). Five where Chinese studies and four Japanese studies. PC resulted in significantly better prognosis in the Chinese studies (HR, 0.61; 95 % CI, 0.47 to 0.80; *P* < 0.01) but not in the Japanese studies (HR, 0.88; 95 % CI, 0.68 to 1.13; *P* = 0.30).

### 1-, 2-, 3-, 5-year survival rates

Nine [9, 17–22, 24, 25], nine [9, 17–22, 24, 25], seven [9, 17, 18, 20–22, 25], six [9, 18, 20, 21, 23, 25] studies reported 1-, 2-, 3-, 5- year survival rates, respectively. There were no significant differences in the 1- and 2-year survival rates between the two study groups (1-year survival rate: RR, 0.81; 95 % CI, 0.60 to 1.09; *P* = 0.17, Additional file 2A; 2-year survival rate: RR, 0.90; 95 % CI, 0.77 to 1.04; *P* = 0.15, Additional file 2B). However, the PC group showed significantly better prognosis for 3- and 5-year survival rates (3-year survival rate: RR, 0.80; 95 % CI, 0.67 to 0.96; *P* = 0.01, Additional file 2C; 5-year survival rate: RR, 0.77; 95 % CI, 0.64 to 0.92; *P* < 0.01, Additional file 2D).

### Response rate to neoadjuvant chemotherapy

Eight studies [9, 11, 17–20, 22, 24] reported the response rates to NAC in 358 patients. The response rate ranged between 33.3 and 70.0 %. In total, 199 patients achieved CR or PR. The overall response rate was 55.6 %.

### Radical resection rate

**Fig. 3:**
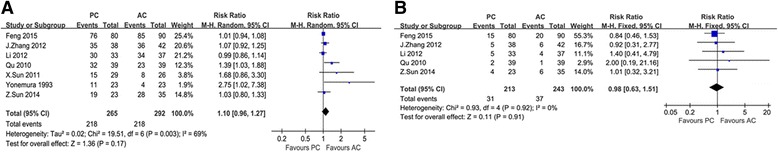
**a** Meta-analysis of Hazard ratio for radical resection rate; **b** Meta-analysis of postoperative complication rate

Seven studies [9, 11, 17–19, 22, 24] reported the radical resection rate. A total of 218 out of 265 patients (82 %) in the PC group and 218 out of 292 (74 %) patients in the AC group received radical resection. Although no significant difference was observed, the PC group showed a trend towards a higher radical resection rate (RR, 1.10; 95 % CI, 0.96 to 1.27; *P* = 0.17, Fig. 3a).

### Total post-operative complication rate

Five studies [9, 11, 19, 22, 24] reported the prevalence of complications. A total of 31 out of the 213 patients in the PC group and 37 out of 243 patients in the AC group suffered postoperative complications. There was no significant difference between two groups (RR, 0.98; 95 % CI, 0.63 to 1.51; *P* = 0.91, Fig. 3b).

### Adverse effects of chemotherapy

Three studies [17, 19, 24] reported adverse effects of chemotherapy in detail. Our meta-analysis indicated that all the adverse effects (including nausea and vomit, gastrointestinal problem, liver toxicity, neurologic effects, leukopenia, thrombocytopenia, and neutropenia) were not significantly different between the two study groups (*P* > 0.05 for all the comparisons, Additional file 3).

### Publication bias

**Fig. 4:**
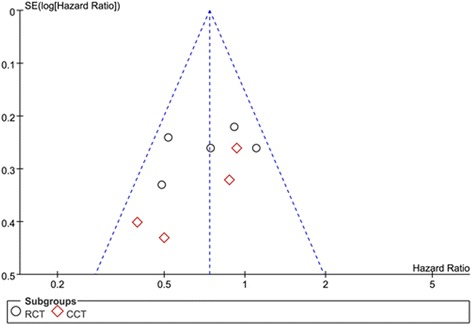
Funnel plot of the studies on hazard ratio for overall survival

A funnel-plot analysis was performed to determine the publication bias on the basis of the measurement of HR for OS (Fig. 4). The analysis indicated that all the studies were within the funnel plot and were distributed symmetrically.

## Discussion

Two methods of chemotherapy delivery, perioperative chemotherapy (PC) and adjuvant chemotherapy (AC) are widely used. However, there is no high level evidence comparing the prognosis and safety between PC and AC. Our meta-analysis that synthesized the results of several smaller studies showed that PC was superior to AC when considering the HR for OS and the 3–, 5-year survival rates. This result indicates that the addition of chemotherapy prior to surgery could provide additional benefits over chemotherapy provided after surgery alone. This may occur because of the effects of NAC in reducing tumor burden and eradicating micrometastatic foci [8]. In this meta-analysis, the response rate to NAC reached 55.6 %. In addition, the radical resection rate was relatively higher in the PC group.

Fluoropyrimidine is the most common and widely accepted chemotherapy drug for GC [26, 27]. However, combination chemotherapy that includes fluoropyrimidine rather than fluoropyrimidine alone is used and recommended by most professionals [4, 28–31]. In our meta-analysis, PC failed to show significant benefits compared with AC in the fluoropyrimidine monotherapy subgroup. However, a significant difference was observed between the two groups in the combination chemotherapy subgroup. This suggests that combination chemotherapy is a better option for NAC in GC and provides significant advantage in relation to fluoropyrimidine monotherapy.

The stage of cancer is another important issue when choosing the treatment method. In the NCCN guideline, for early-stage GC, PC is not routinely recommended [6]. To better understand this issue, we considered the tumor stages reported in the studies included in this meta-analysis. In addition, in almost all the studies included, the majority, if not all, of the patients were at advanced stages. However, the RCT by Nio et al. [20] was an exception. Of the 295 patients evaluated in the RCT, 170 patients were stage 1, and this subgroup failed to show any advantage of PC over AC. On the other hand, when we redid the meta-analysis excluding stage 1 patients and only including stage 2 and 3 patients, the survival benefits of PC became more significant (HR 0.68; 95 % CI, 0.56 to 0.83; *P* < 0.01). Therefore, the conclusion that “perioperative chemotherapy was superior to adjuvant chemotherapy in the survival benefits” seems to be more suitable for advanced GC.

The results of two large European RCTs [4, 32], which compared PC and surgery alone, established PC as another alternative option for GC. However, no European studies have compared PC and AC. Furthermore, all 11 studies included in this meta-analysis were from Asian countries (China and Japan). We performed a subgroup analysis for these two countries and the results indicated that the Chinese group showed an advantage compared with the Japanese group. This observation may be explained by the fact that Nio et al. [20] included many early-stage patients and most Japanese studies used fluoropyrimidine monotherapy as their NAC method. This suggests that Japanese groups should give more consideration to the use of combination chemotherapy, especially in the pre-operative setting.

Another potential advantage of perioperative chemotherapy is that it increases the likelihood that patients will receive at least part of their planned systemic chemotherapy regimen. For example, Yonemura [17] reported that patients in PC group received relative more courses of regimen in fact. In that study, patients in PC and AC received an average of 2.9 and 2.3 courses of regimen respectively. However, this issue is not reported by the others studies. We believe that studies should pay more attention to this matter in the future.

Safety is always of the utmost concern in clinical practice. Because fibrosis, tissue edema and toxicity may result from chemotherapy [11, 33], there is a concern that the addition of chemotherapy prior to surgery may increase the risk during surgery as well as increase complication rates and adverse effects [34]. In our study, the complication rates and adverse effects during PC were similar to those of AC. Postoperative complication rates were also consistent with the studies that evaluated the effects of NAC (without the restrictions of AC administration) [35, 36]. However, only three studies reported adverse effects, and these studies were not enough to draw a solid conclusion. More investigations are needed to evaluate the side effects of PC.

To the best of our knowledge, this is the first meta-analysis that compares PC with AC in GC. In our meta-analysis, we included 11 studies, five of which were RCTs, and found that PC was superior to AC in the survival effects without compromising safety. In addition, combination chemotherapy was a better option in the pre-operative setting over monotherapy. A subgroup analysis involving three studies [10] in a previous meta-analysis evaluated the effects of NAC in GC and found similar survival benefits. Several limitations of our study should be considered. First, we included some retrospective studies, which may influence the statistical power. Second, all studies were from Asia and limits its generalizability for GC patients worldwide. Third, only a few studies compared the adverse effects of chemotherapy, which did not allow us to draw conclusions about the safety of PC.

## Conclusion

Perioperative chemotherapy provides a survival advantage over adjuvant chemotherapy for GC patients, especially for the patients with advanced GC. In addition, combination chemotherapy is a better option for neoadjuvant chemotherapy regimen over monotherapy.

## Additional files

Additional file 1: Concrete chemotherapy regimens used in included studies. (DOCX 22 kb) Additional file 2: (A) Meta-analysis of 1 year survival rate; (B) Meta-analysis of 2 year survival rate; (C) Meta-analysis of 3 year survival rate; (D) Meta-analysis of 5 year survival rate. (TIF 457 kb) Additional file 3: meta-analysis of chemotherapy adverse effects. (A) Nausea and vomit, (B) gastrointestinal problem, (C) liver toxicity, (D) neurologic effects, (E) leukopenia, (F) thrombocytopenia, (G) neutropenia. (TIF 507 kb)

## Abbreviations

AC: Adjuvant Chemotherapy
CCTs: Clinical Controlled Trials
CIs: Confidence Intervals
CR: Complete Response
GC: Gastric Cancer
HR: Hazard Ratio
NAC: Neoadjuvant Chemotherapy
OS: Overall Survival
PC: Perioperative Chemotherapy
PR: Partial Response
RCTs: Randomized Controlled Trials
RR: Risk Ratio
