# Supplementary material for: Which is better for gastric cancer patients, perioperative or adjuvant chemotherapy: a meta-analysis
Source: BMC Cancer. 2016 Aug 12;16:631. doi: 10.1186/s12885-016-2667-5 (PMC4983077; doi:10.1186/s12885-016-2667-5)
Supplement: Additional file 1: — Concrete chemotherapy regimens used in included studies. (DOCX 22 kb) [file 12885_2016_2667_MOESM1_ESM.docx]

Additional file 1. Concrete chemotherapy regimens used in included studies

| Study | Year | NAC regimen in PC group | Postoperative chemotherapy regimen in PC group | Postoperative chemotherapy regimen in AC group |
| --- | --- | --- | --- | --- |
| Yonemura | 1993 | PMUE: UFT 400mg orally daily; cisplatin 75 mg/m^2^ iv, d1; mitomycin-C 10 mg, iv, d1; Etoposide 50 mg, d3, d4, d5; each cycle 3 weeks. | PMUE: UFT 400mg orally daily; cisplatin 75 mg/m^2^ iv, d1; mitomycin-C 10 mg, iv, d1; Etoposide 50 mg, d3, d4, d5; each cycle 3 weeks. | PMUE: UFT 400mg orally daily; cisplatin 75 mg/m^2^ iv, d1; mitomycin-C 10 mg, iv, d1; Etoposide 50 mg, d3, d4, d5; each cycle 3 weeks. |
| Kobayashi | 2000 | 5'-DFUR: 610mg/m^2^/day more than 10 days, orally | 5'-DFUR+MMC: MMC iv d1,2; 5'-DFUR, more than 2 years, orally | 5'-DFUR+MMC: MMC iv d1,2; 5'-DFUR, more than 2 years, orally |
| Nio | 2004 | UFT: UFT 8 mg/kg/d for the patients under 70 years and 6 mg/kg/d for those over 71 years, orally, usually more than two weeks | UFT or FPEPIR+UFT: Stage 1-3 patients UFT for 1-3 year; Stage 4 patients received 1-4 courses of CDDP, 5-FU and Epirubicin (FPEPIR regimen) and then received oral UFT | UFT or FPEPIR+UFT: Stage 1-3 patients UFT for 1-3 year; Stage 4 patients received 1-4 courses of CDDP, 5-FU and Epirubicin (FPEPIR regimen) and then received oral UFT |
| Qul | 2010 | PTX+FOLFOX4: Paclitaxel 135 mg/m^2^, d1; oxaliplatin 85 mg/m^2^, d1; folinic acid 200 mg/m^2^, d1, d2; 5-FU 400 mg/m^2^, iv d1, d2; 600 mg/m^2^,civ 22 h, d1, d2; 3 cycles, each cycle 2 weeks. | PTX+FOLFOX4 or ECF: Patients with PD, give ECF regimen (Cisplatin, 5-FU, Epirubicin). Otherwise, continue 3 cycles of FOLFOX4 as NAC | PTX+FOLFOX4: Paclitaxel 135 mg/m^2^, d1; oxaliplatin 85 mg/m^2^, d1; folinic acid 200 mg/m^2^, d1, d2; 5-FU 400 mg/m^2^, iv d1, d2; 600 mg/m^2^,civ 22 h, d1, d2; 6 cycles, each cycle 2 weeks. |
| X.Sun | 2011 | DCF: docetaxel,cisplatin 75 mg/m^2^ iv d1; 5-FU 500mg/m^2^ iv d1-3, leucovorin 200 mg/m^2^ iv before 5-FU, then by civ over 2h on d1-d5, 3 cycles, each cycle 3 weeks. | DCF: docetaxel,cisplatin 75 mg/m^2^ iv d1; 5-FU 500mg/m^2^ iv d1-3, leucovorin 200 mg/m^2^ iv before 5-FU, then by civ over 2h on d1-d5, 3 cycles, each cycle 3 weeks. | DCF: docetaxel,cisplatin 75 mg/m^2^ iv d1; 5-FU 500mg/m^2^ iv d1-3, leucovorin 200 mg/m^2^ iv before 5-FU, then by civ over 2h on d1-d5, 6 cycles, each cycle 3 weeks. |
| Z.Sun | 2014 | FOLFOX4: oxaliplatin 85 mg/m^2^, d1; folinic acid 200 mg/m^2^, d1, d2; 5-FU 400 mg/m^2^, iv d1, d2; 600 mg/m^2^,civ 22 h, d1, d2; 2-6 cycles, each cycle 2-3 weeks. | FOLFOX: 1-6 cycles, combination of oxaliplatin, folinic acid and 5-FU. (concrete not mentioned) | FOLFOX: 1-6 cycles, combination of oxaliplatin, folinic acid and 5-FU. (concrete not mentioned) |
| Feng | 2015 | SOX: S-1 orally twice a day for a total of 80 mg/m^2^ from d1 to d14, and oxaliplatin 130 mg/m^2^, iv, d1, 2-4 cycles, each cycle 3 weeks | SOX: S-1 orally twice a day for a total of 80 mg/m^2^ from d1 to d14, and oxaliplatin 130 mg/m^2^, iv, d1, 4-6 cycles, each cycle 3 weeks | SOX: S-1 orally twice a day for a total of 80 mg/m^2^ from d1 to d14, and oxaliplatin 130 mg/m^2^, iv, d1, 8 cycles, each cycle 3 weeks |
| Li | 2012 | FOLFOX: oxaliplatin 130 mg/m^2^, iv, d1; folinic acid 400 mg/m^2^, iv, d1; followed by 5-FU 400 mg/m^2^ iv; 5-FU 2,500 mg/m^2^ civ 22h; 2-4 cycles, each cycle 3 weeks | FOLFOX: oxaliplatin 130 mg/m^2^, iv, d1; folinic acid 400 mg/m^2^, iv, d1; followed by 5-FU 400 mg/m^2^ iv; 5-FU 2,500 mg/m^2^ civ 22h; 6 cycles, each cycle 3 weeks | FOLFOX: oxaliplatin 130 mg/m^2^, iv, d1; folinic acid 400 mg/m^2^, iv, d1; followed by 5-FU 400 mg/m^2^ iv; 5-FU 2,500 mg/m^2^ civ 22h; 6 cycles, each cycle 3 weeks |
| J.Zhang | 2012 | mFOLFOX7: calcium folinate 400 mg/m^2^, iv, d1; oxaliplatin 100 mg/m^2^, iv, d1; 5-FU 2400 mg/m^2^ civ 46h, d2; 4 cycles; each cycle about 2 weeks | mFOLFOX7 or mECF: Patients with PD, give mECF regimen. Otherwise, continue 12 cycles of mFOLFOX7 as NAC | mFOLFOX7: calcium folinate 400 mg/m^2^, iv, d1; oxaliplatin 100 mg/m^2^, iv, d1; 5-FU 2400 mg/m^2^ civ 46h, d2; 12 cycles; each cycle took about 2 weeks |
| Nishioka | 1982 | 5-FU: 500mg for 10days, orally | 5-FU and MMP: 5-FU more than 5000mg; MMC 40mg | 5-FU and MMP: 5-FU more than 5000mg; MMC 40mg |
| C.Zhang | 2004 | FAP or FMP: FAP (5-FU 1.0 g; ADM 30-50 mg; DDP 40-60 mg); FMP (5-FU 1.0 g; MMC 8-10 mg; DDP 40-60 mg); 1-2 times; interval time 10-14d | FAP or FMP: FAP (5-FU 1.0 g; ADM 30-50 mg; DDP 40-60 mg); FMP (5-FU 1.0 g; MMC 8-10 mg; DDP 40-60 mg); 1-2 times; interval time 10-14d | FAP or FMP: FAP (5-FU 1.0 g; ADM 30-50 mg; DDP 40-60 mg); FMP (5-FU 1.0 g; MMC 8-10 mg; DDP 40-60 mg) |
