# Supplementary figures and images for: Which is better for gastric cancer patients, perioperative or adjuvant chemotherapy: a meta-analysis
Source: BMC Cancer. 2016 Aug 12;16:631. doi: 10.1186/s12885-016-2667-5 (PMC4983077; doi:10.1186/s12885-016-2667-5)

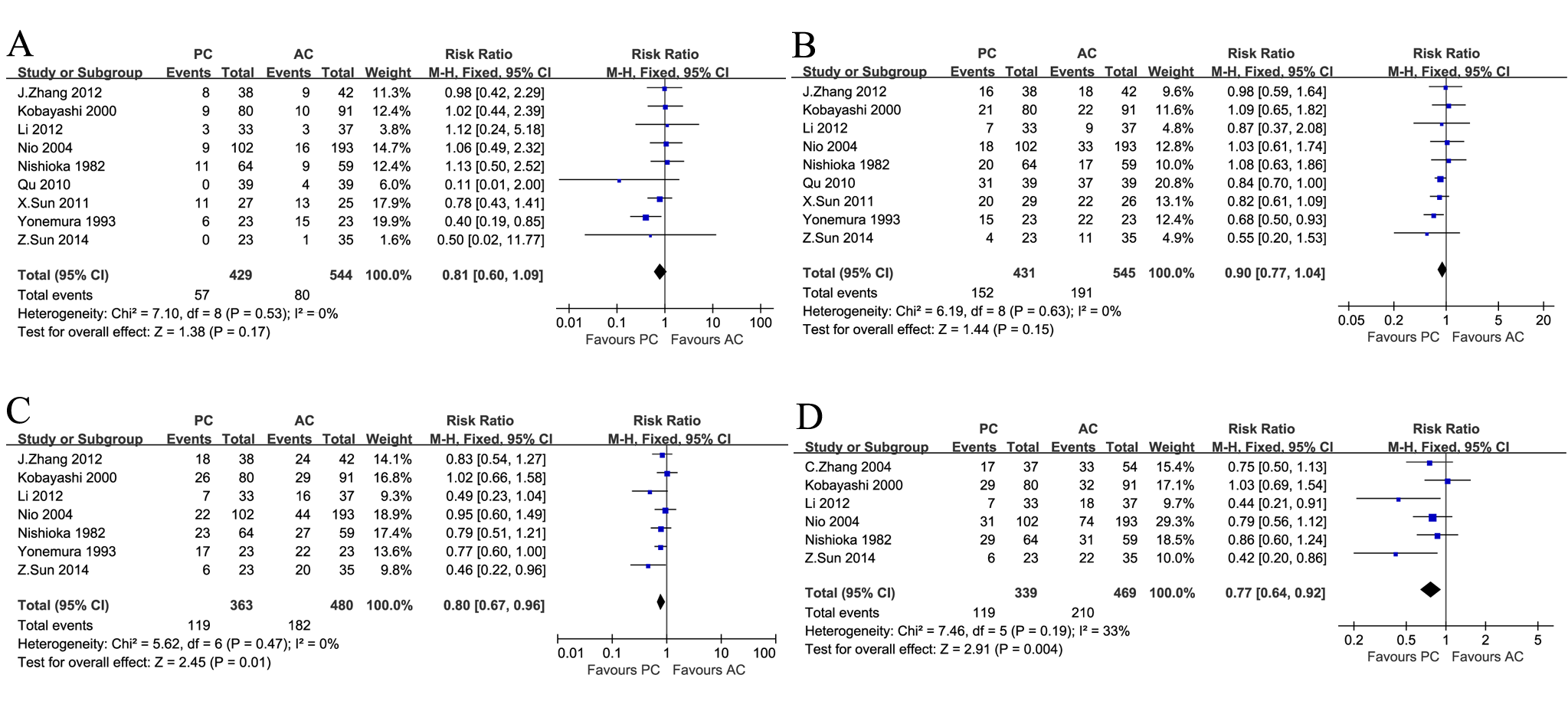

Supplement: Additional file 2: — (A) Meta-analysis of 1 year survival rate; (B) Meta-analysis of 2 year survival rate; (C) Meta-analysis of 3 year survival rate; (D) Meta-analysis of 5 year survival rate. (TIF 457 kb) [file 12885_2016_2667_MOESM2_ESM.tif]

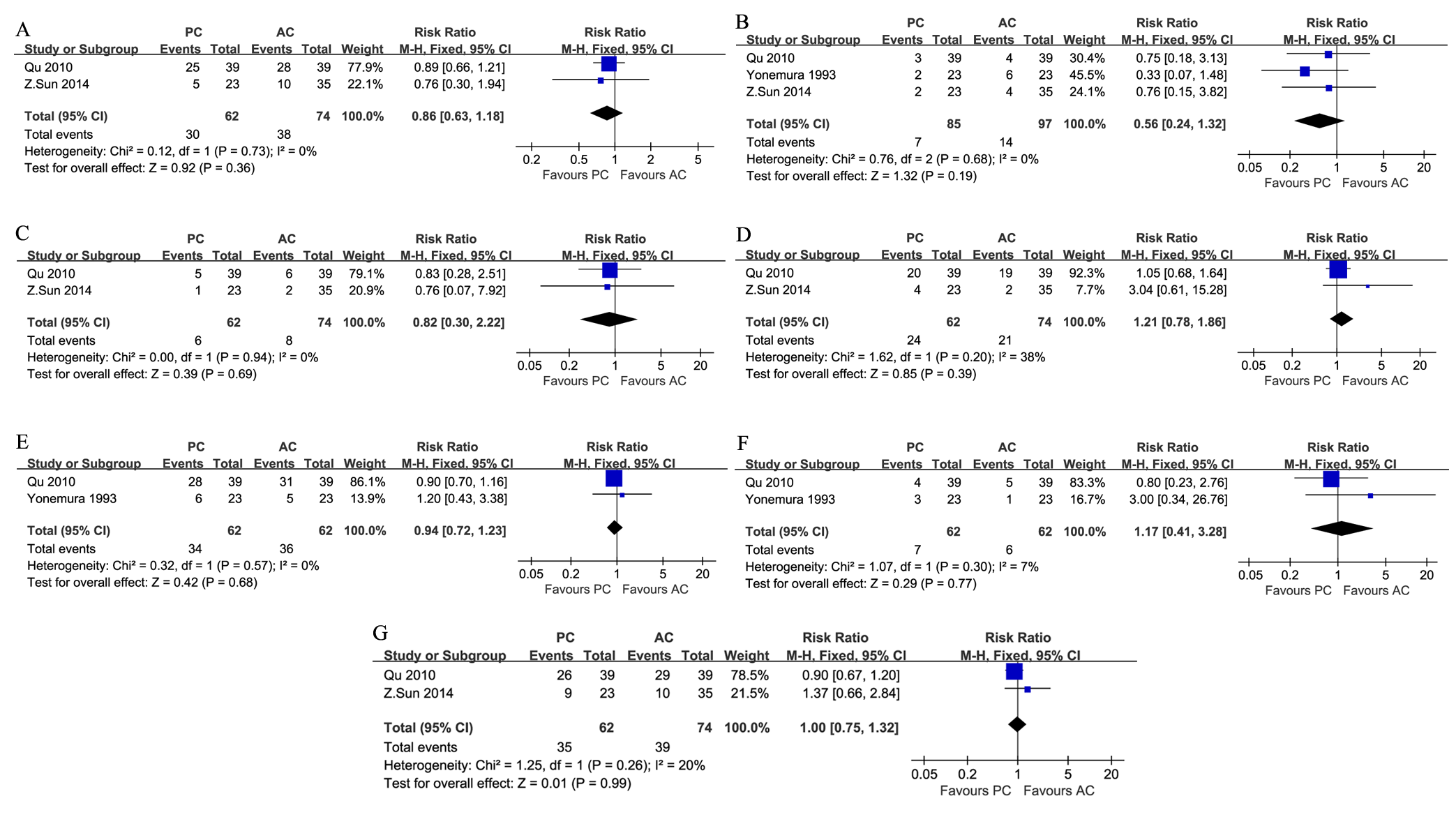

Supplement: Additional file 3: — meta-analysis of chemotherapy adverse effects. (A) Nausea and vomit, (B) gastrointestinal problem, (C) liver toxicity, (D) neurologic effects, (E) leukopenia, (F) thrombocytopenia, (G) neutropenia. (TIF 507 kb) [file 12885_2016_2667_MOESM3_ESM.tif]
